# Hypervirulent FAdV-4 infection induces activation of the NLRP3 inflammasome in chicken macrophages

**DOI:** 10.1016/j.psj.2021.101695

**Published:** 2021-12-31

**Authors:** Baiyu Wang, Huifang Guo, Qilong Qiao, Qing Huang, Panpan Yang, Congcong Song, Mingzhen Song, Zeng Wang, Yongtao Li, Yuhe Miao, Jun Zhao

**Affiliations:** ⁎ College of Veterinary Medicine, Henan Agricultural University, Zhengzhou 450046, China; † Fujian Shengwei Biotech Co., Ltd., Nanping 354100, China

**Keywords:** fowl adenovirus serotype 4, NLRP3 inflammasome, Caspase-1, interleukin-1 beta, chicken macrophage

## Abstract

Fowl adenovirus serotype 4 (**FAdV-4**) is the primary causative agent of hepatitis–hydropericardium syndrome (**HHS**) causing great economic losses to the world poultry industry. The exact factors responsible for the pathogenesis of hypervirulent FAdV-4 have not been completely elucidated. Hypervirulent FAdV-4 infection induces inflammatory damages in accompany with a high level of proinflammatory interleukin-1 beta (**IL-1β**) secretion in a variety of organs. Investigation of the mechanisms underlying hypervirulent FAdV-4-induced IL-1β secretion would contribute to understanding of the pathogenesis of FAdV-4. Here, we investigated whether FAdV-4 infection activates NLRP3 inflammasome in chicken macrophage cell line HD11. The results showed that stimulation of HD11 with hypervirulent FAdV-4 induced NLRP3- and Caspase-1-dependent secretion of IL-1β. Genetic knockdown of NLRP3 or Caspase-1 expression, a critical component of inflammasome, significantly downregulated IL-1β expression, indicating that activation of the NLRP3 inflammasome contributed to the FAdV-4-induced IL-1β secretion. Moreover, ATP signaling and potassium efflux were involved in the process of NLRP3 inflammasome activation. Our data indicated that hypervirulent FAdV-4 infection induces the activation of NLRP3 inflammasome and followed by massive secretion of IL-1β of macrophages, which thereby contribute to the inflamed lesion of tissues.

## INTRODUCTION

Fowl adenovirus serotype 4 (**FAdV-4**) belongs to fowl aviadenovirus species C of *Aviadenovirus* genus, *Adenoviridae* family (Shah et al., 2017; Niu et al., 2019). Pathogenic FAdV-4 is the predominant etiological agent of hepatitis-hydropericardium syndrome (**HHS**) which mainly affects 3 to 6-wk-old broiler chickens with up to 80% mortality (Dahiya et al., 2002; Ye et al., 2016; Li et al., 2017). HHS induced by a novel genotype of FAdV-4 becomes prevalence in China since May 2015, causing huge economic losses (Sun et al., 2019; Yu et al., 2019). Previous research indicated that hypervirulent FAdV-4 infection induces severe inflammatory histopathological damages in multiple organs of infected chickens including the accumulation of pericardial effusion, severe depletion of lymphocytes in the spleen and thymus, necrosis of hepatocytes, pulmonary and renal edema, etc. (Zhao et al., 2015; Liu et al., 2016; Pan et al., 2017; Li et al., 2018b; Pan et al., 2018; Yu et al., 2018; Zhang et al., 2018). However, the exact factors responsible for the FAdV-4 pathogenesis remain unknown.

Pro-inflammatory cytokine interleukin-1 beta (**IL-1β**) is an important signaling molecule that mediates inflammatory responses and participates in the process of monocytes differentiation and pathogen removal (Bent et al., 2018). However, excessive accumulation of IL-1β might cause inflammatory damages and acute death of the host (Dinarello and van der Meer, 2011; Chen et al., 2018; Wang and Zhao, 2019). Hypervirulent FAdV-4 infection induces significant upregulations of IL-1β in the primary viral targeting organs such as liver, spleen, and the bursa of Fabricius (Li et al., 2018a; Meng et al., 2019; Zhao et al., 2020). Significantly increased expression of IL-1β was also noticed in the chicken hepatocellular carcinoma cell line infected with FAdV-4 (Niu et al., 2018). Investigation of the mechanisms underlying hypervirulent FAdV-4-induced IL-1β expression will contribute to understand the pathogenesis of FAdV-4. Nod-like receptor family pyrin domain containing 3 (**NLRP3**) inflammasome has a major part to play in the immune responses during viral infections as it senses the invading pathogen-associated molecular patterns (**PAMPs**) (Wang et al., 2016; Gao et al., 2020). NLRP3 inflammasome is formed of a sensor protein NLRP3, an adaptor-apoptosis-associated speck-like protein containing a caspase recruitment domain (**ASC**), and a downstream effector pro-Caspase-1 (Srinivasula et al., 2002). Upon activation, NLRP3 inflammasome triggers the conversion of pro-Caspase-1 into the active form, Caspase-1, which subsequently cleaves pro-IL-18 and pro-IL-1β into proinflammatory cytokine IL-18 and IL-1β (Wei et al., 2021). Previous studies have showed that cytokine storm induced by excessive activation of NLRP3 inflammasome leads to tissue damage (Compan et al., 2012; Lin et al., 2019). Activation of NLRP3 inflammasome and upregulation of the secretion of IL-1β in mice and human macrophages by viral infection have been proved previously (Wang et al., 2016; Ye et al., 2021). However, whether NLRP3 inflammasome mediates the expression of IL-1β in chicken macrophages upon stimulation of hypervirulent FAdV-4 is still unclear. In the present study, FAdV-4 infection-induced NLRP3 inflammasome activation in chicken macrophages and the possible activation mechanism were investigated.

## MATERIALS AND METHODS

### Virus, Cells and Reagents

Hypervirulent FAdV-4 strain CH/HNJZ/2015 (GenBank ID: KU558760) was obtained previously by virus isolation from chicken flocks suffering from the hepatitis–hydropericardium syndrome (Liu et al., 2016). Chicken macrophage cell line HD11 originated from chicken bone marrows was cultured in RPMI medium supplemented with 10% fetal bone serum (**FBS**) at 37°C, 5% CO_2_. Recombinant plasmids pCAGGS-NLRP3 and pCAGGS-Caspase-1 were constructed by cloning chicken NLRP3 and Caspase-1 gene into vector pCAGGS-(HA). Small-interfering RNA (**siRNA**) against NLRP3 components, siNLRP3 (sense: 5’-UUGCAAAGGACGUGAAUAUTT-3’, antisense: 5’-AUAUUCACGUCCUUUGCAATT-3’), siCaspase-1 (sense: 5’-CCUUUAACAGUGACUUGCAGGAGAU-3’, antisense: 5’-AUCUCCUGCAAGUCACUGUUAAAGG-3’) and negative control siNC (sense: 5’-UUCUCCGAACGUGUCACGUTT-3’, antisense: 5’-ACGUGACACGUUCGGAGAATT-3’) were designed and synthesized by Sangon Biotech (Shanghai, China) Co., Ltd.

### Virus Infection and Cell Stimulation

HD11 cells were seeded in 12-well plates (8 × 10^5^ cells / well) overnight before different treatments. First, to determine whether ATP is involved in IL-1β secretion during FAdV-4 infection, the cells were inoculated with FAdV-4 (MOI of 5) and incubated for 4 h at 37°C, and 0 or 5 mM ATP was then added. The supernatants were harvested at various timepoints post-ATP stimulation to determine the secretion level of IL-1β by ELISA.

To test whether FAdV-4-induced IL-1β secretion is mediated by NLRP3 inflammasome, the HD11 cells were transfected with either siRNA against NLRP3 inflammasome components (40 pmol of siCaspase-1, siNLRP3, or siNC) or 4 μg of recombinant plasmids pCAGGS-NLRP3, pCAGGS-Caspase-1 or the empty plasmid pCAGGS-(HA) using Lipofectamine2000 transfection reagent (Thermo Fisher Scientific Inc., Waltham, MA) according to the manufacturer's instructions.

In order to re-confirm whether FAdV-4-induced IL-1β release is dependent on Caspase-1, HD11 cells were incubated with 20 μM of Ac-YVAD-CHO (Merck, 400010-1MGCN, Darmstadt, Germany) for 1 h, and Ac-YVAD-CHO was then removed before FAdV-4 and ATP stimulation.

HD11 cells were also treated with either 130 mM or 5 mM KCl for 5 min to evaluate the possible role of potassium efflux in the inflammasome activation by FAdV-4 infection.

At 36 h post-transfection of siRNA or recombinant plasmids, or after Ac-YVAD-CHO or KCl treatments, the cells were treated with FAdV-4 at MOI of 5 for 4 h, and then with 5-mM ATP for 1 h. Cell-free supernatants were harvested for detection of IL-1β by ELISA, and cell lysates were prepared for Western blot analysis or Caspase-1 activity assay.

### Caspase-1 Activity Assay

Caspase-1 activity in the lysates of Ac-YVAD-CHO-treated cells was detected by Caspase-1 Activity Assay Kit (Beyotime, C1102, Shanghai, China) according to the manufacturer's protocols. Each sample was tested in triplicates. The lysates were incubated with 2mM of Ac-YVAD-pNA at 37°C for 12 h before OD_405_ measurement. The amount of pNA produced by Caspase-1 catalysis was calculated by a standard curve method provided by the kit.

### Chicken IL-1β ELISA

IL-1β secretion level was evaluated by using chicken IL-1β ELISA kit (Cloud-clone Corp., SEA563Ga, Houston, TX) according to the manufacturer's protocols. After the HD11 cells were stimulated with FAdV-4 or FAdV-4 + ATP under different conditions, cell-free supernatants were collected and tested in triplicates.

### Western Blot Analysis

HD11 cells were lysed on ice with Western&IP lysate buffer (Beyotime Biotechnology Inc., Shanghai, China). The concentration of protein in the lysates was determined by the bicinchoninic acid (**BCA**) method. Same amount of total protein samples was subjected to SDS-PAGE, and then transferred to nitrocellulose (**NC**) membranes. The NC membranes were incubated with different primary antibodies, such as rabbit anti-NLRP3 (1:400, Wanleibio, WLH3383, Shenyang, China), rabbit anti-cleaved-Caspase-1 (1:400, Wanleibio, WL03450, Shenyang, China) or mouse anti-Tublin (1:1,000, Beyotime, AT819, Shanghai, China). Then, NC membranes were probed with HRP-conjugated goat anti-rabbit IgG (1:3,000, Proteintech, SA00001-2, Chicago, IL) or goat anti-mouse IgG (1:3,000, Proteintech, SA00001-1, Chicago, IL) and ECL reagent on Amersham Imager 680, and the intensity of protein bands was quantified using ImageJ software.

### Statistical Analysis

The present study utilized GraphPad Prism 5.0 for statistical analysis of IL-1β secretion level in different treatment groups. Significant difference analysis was performed using the *t*-test. A *P*-value < 0.05 was determined to be statistically significant.

## RESULTS

### Exogenous ATP is Required for IL-1β Secretion in Chicken Macrophage Cells Induced by Hypervirulent FAdV-4 Infection

**Figure 1:**
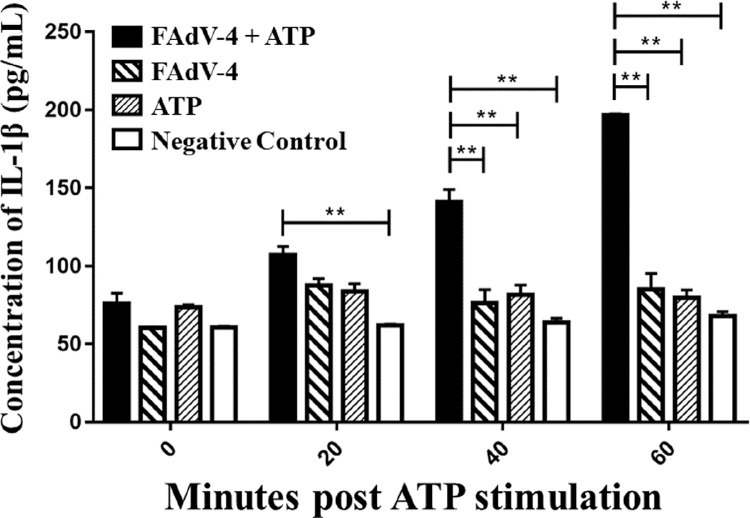
ATP is necessary for the IL-1β secretion induced by hypervirulent FAdV-4 (*P < 0.05; **P< 0.01; ***P < 0.001).

To determine the role of ATP in IL-1β secretion induced by hypervirulent FAdV-4 infection, HD11 cells were stimulated by hypervirulent FAdV-4 for 4 h followed with ATP treatment for 20, 40, and 60 min, respectively. The cell-free supernatant was collected and the secretion level of IL-1β was detected by ELISA. The results demonstrated that compared to the FAdV-4 only group and the ATP only group, FAdV-4 + ATP induced significantly higher level of IL-1β secretion. In addition, the secretion level was positively correlated with the incubation time (Figure 1).

### FAdV-4-Induced IL-1β Secretion is Mediated by NLRP3 Inflammasome

**Figure 2:**
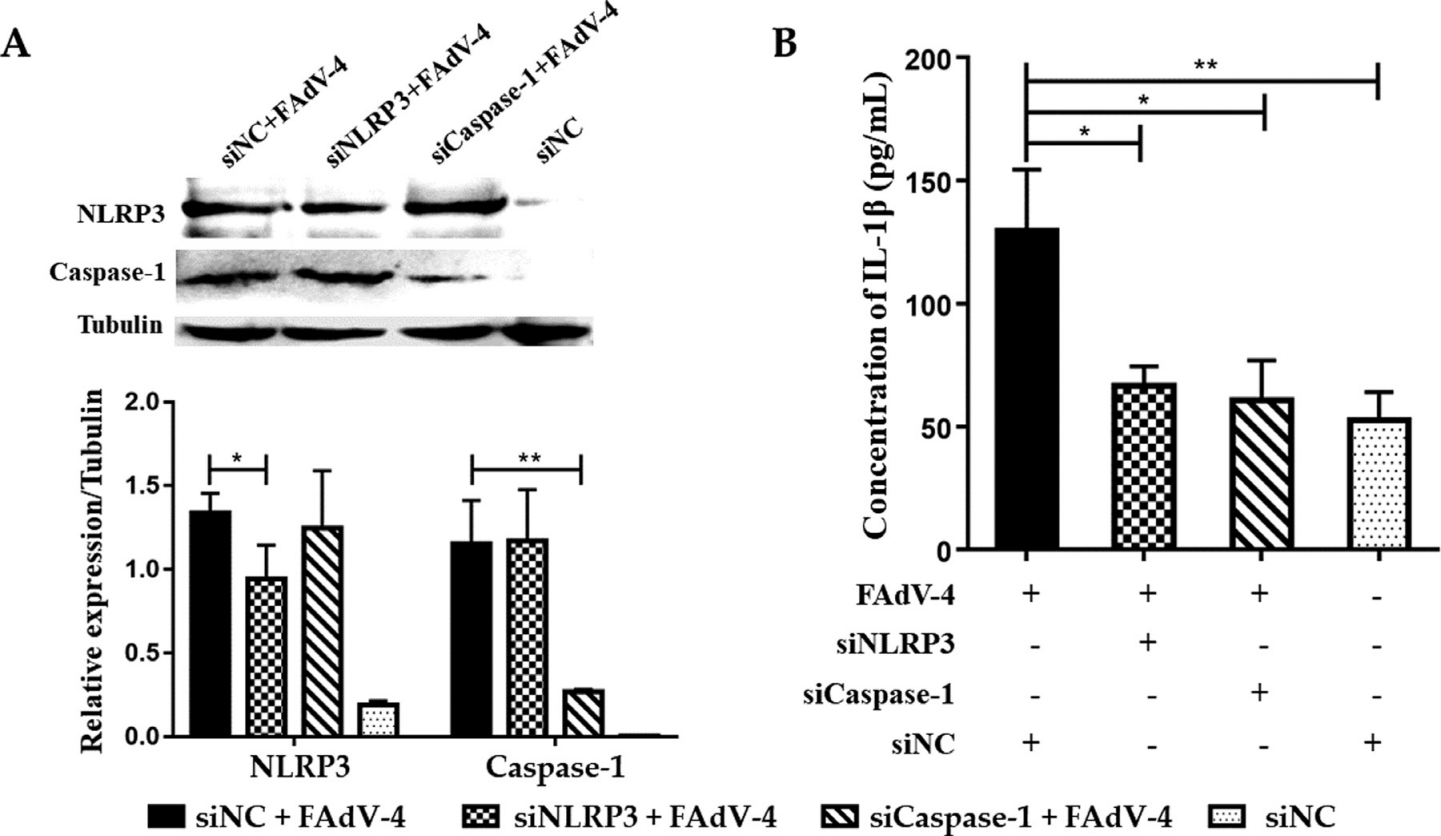
IL-1β secretion level after knock-down of NLRP3 and Caspase-1 in HD11 cells. (A) Protein expression levels of NLRP3 and Caspase-1 in HD11 cells were reduced after knock-down by siRNA. (B) The secretion of IL-1β was significantly reduced in HD11 cells with NLRP3 and Caspase-1 knocked down and FAdV-4 and ATP stimulation (*P < 0.05; **P < 0.01).

**Figure 3:**
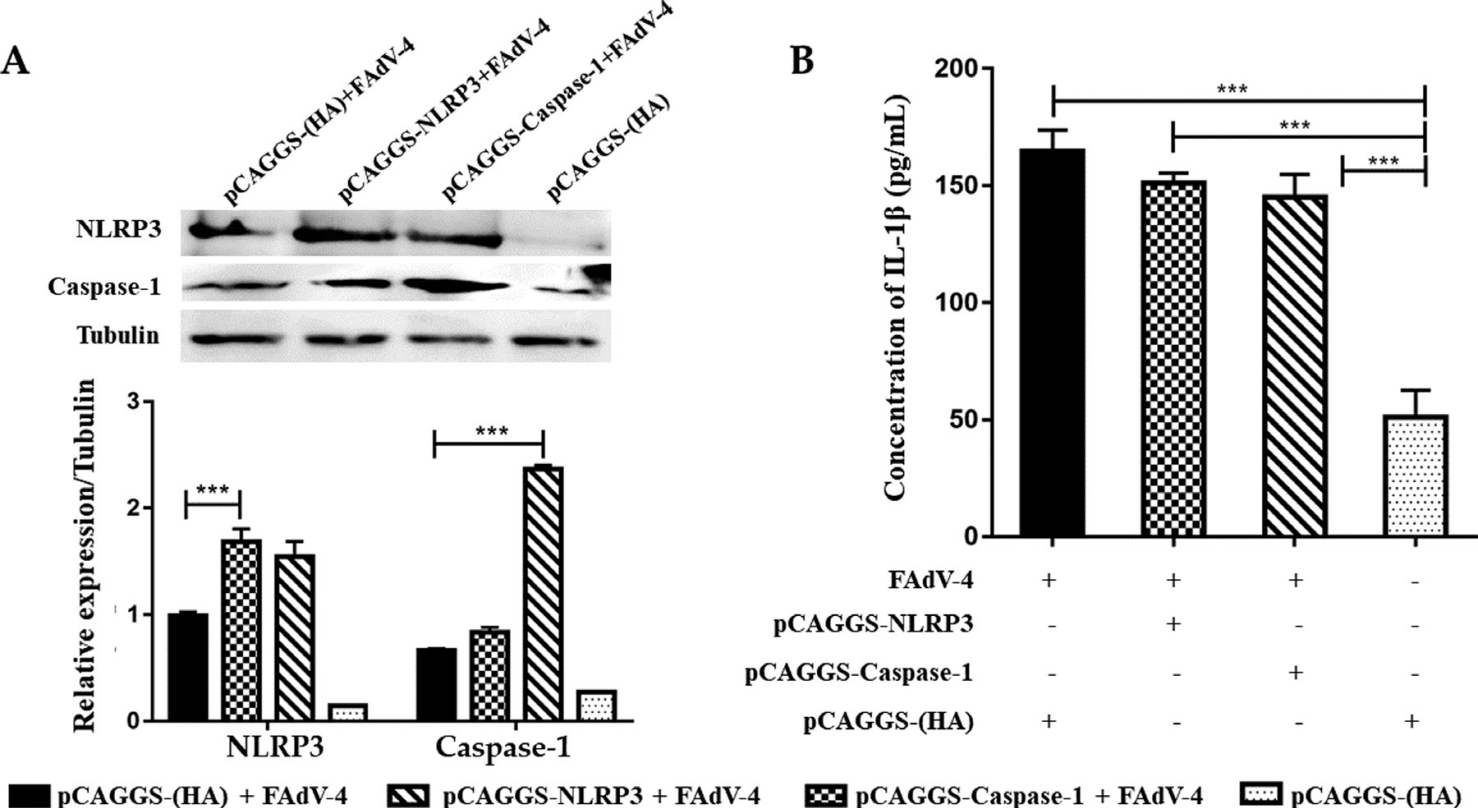
IL-1β secretion level in HD11 cells overexpressed with NLRP3 and Caspase-1. (A) Protein expression levels of NLRP3 and Caspase-1 in HD11 cells were increased after overexpression by transfection of recombinant plasmids. (B) Significant upregulation of IL-1β was observed in the pCAGGS-NLRP3 and pCAGGS-Caspase-1 plasmids transfected plus FAdV-4 stimulated cells compared to that of the control plasmid pCAGGS-(HA) transfected cells (***P < 0.001).

To test whether FAdV-4-induced IL-1β release is mediated by NLRP3 inflammasome, HD11 cells were transfected with either siRNA against NLRP3 inflammasome components or recombinant plasmids overexpressing NLRP3 inflammasome components. The expression of NLRP3 and Caspase-1 was upregulated in HD11 cells treated with hypervirulent FAdV-4 plus siNC (Figure 2A). Compared to the siNC + FAdV-4 treated cells, the expression of NLRP3 and IL-1β was significantly reduced in the HD11 cells treated with siNLRP3 + FAdV-4 (*P* < 0.05; Figures 2A and 2B). Similarly, the expression levels of cleaved-Caspase-1 and IL-1β in the siCaspase + FAdV-4 group were significantly downregulated (*P* < 0.05; Figures 2A and 2B). HD11 cells transfected with the recombinant plasmids overexpressing NLRP3 and Caspase-1 demonstrated elevated expression of NLRP3 and Caspase-1, respectively (Figure 3A). The expression of IL-1β was significantly higher in the FAdV-4 only stimulated cells, pCAGGS-NLRP3 and pCAGGS-Caspase-1 plasmids transfected plus FAdV-4 stimulated cells compared to that in the control plasmid pCAGGS-(HA) transfected cells (*P* < 0.001). However, the difference of the IL-1β secretion level among the FAdV-4 only stimulated cells, pCAGGS-NLRP3 and pCAGGS-Caspase-1 plasmids transfected plus FAdV-4 stimulated cells did not reach significant (Figure 3B).

**Figure 4:**
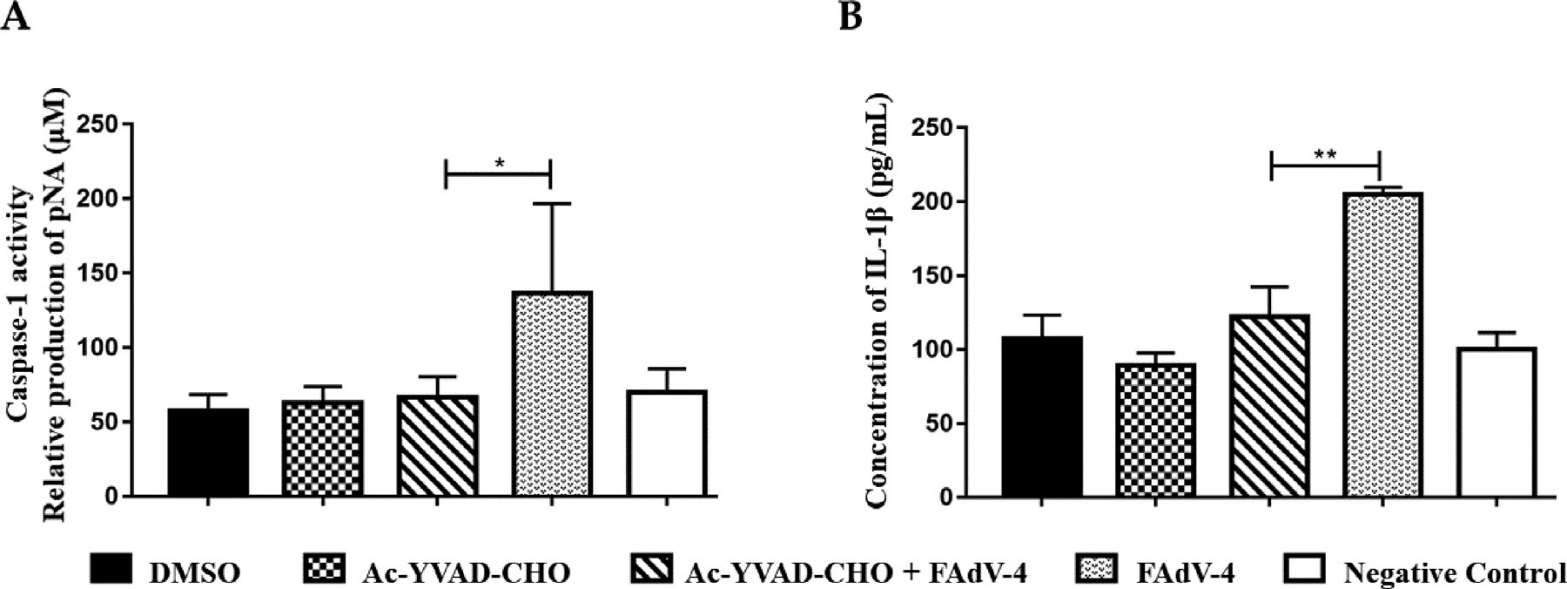
The expression level of IL-1β in HD11 cells with inhibited Caspase-1 activity. (A) Caspase-1 inhibitor Ac-YVAD-CHO effectively inhibited the activity of Caspase-1 in HD11 cells. (B) IL-1β secretion level was significantly decreased in the HD11 cells incubated with Ac-YVAD-CHO (*P < 0.05; **P < 0.01).

In order to reconfirm that FAdV-4-induced IL-1β secretion is directly mediated by Caspase-1, HD11 cells were incubated with Ac-YVAD-CHO, the activity inhibitor of Caspase-1, before FAdV-4 stimulation. Compared to the FAdV-4 only stimulated cells, the Caspase-1 activity in the Ac-YVAD-CHO and FAdV-4 stimulated cells was significantly suppressed (*P* < 0.05; Figure 4A) and the IL-1β secretion was significantly downregulated (*P* < 0.01; Figure 4B).

### K^+^ Efflux Involves in the NLRP3 Inflammasome Activation by Hypervirulent FAdV-4

**Figure 5:**
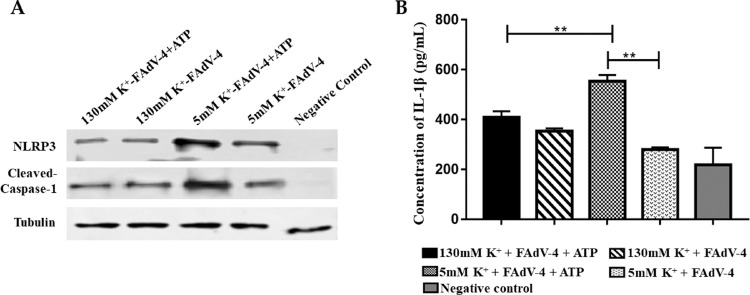
Effects of K^+^ efflux on the activation of NLRP3 inflammasome in FAdV-4 infected HD11 cells. (A) The protein expressions of NLRP3 and cleaved-caspase-1 were reduced in HD11 cells treated with 130 mM KCl, (B) The secretion level of IL-1β was significantly downregulated in HD11 cells treated with 130-mM KCl and ATP and in HD11 cells treated with 5-mM KCl without ATP (***P < 0.001).

To investigate the activation mechanism of NLRP3 by FAdV-4, HD11 cells were stimulated by hypervirulent FAdV-4 and treated with either 5 mM KCl or 130 mM KCl. The results indicated that NLRP3, cleaved-Caspase-1, and IL-1β in HD11 cells treated with 130 mM-KCl + ATP were significantly downregulated compared to the cells treated with 5mM-KCl + ATP (*P* < 0.01; Figures 5-A and 5B). On the other hand, NLRP3, cleaved-Caspase-1, and IL-1β expressions in HD11 cells treated with the 5 mM-KCl only and 130 mM-KCl only were similar (Figures 5A and 5B), indicating the indispensable role of ATP for NLRP3 activation. HD11 cells stimulated with FAdV-4 under the presence of high concentration of extracellular K^+^ impeded the function of ATP to active NLRP3 and process pro-Caspase-1 and pro-IL-1β, indicating that K^+^ efflux induces intracellular signals in chicken macrophages that activate NLRP3 inflammasome.

## DISCUSSION

Hepatitis–hydropericardium syndrome in chickens infected with hypervirulent FAdV-4 has been causing tremendous economic losses to the world poultry industry. Hypervirulent FAdV-4 infection induces inflammatory damage in many tissues accompanying high level secretion of the proinflammatory cytokine IL-1β (Li et al., 2018a; Niu et al., 2018; Meng et al., 2019; Zhao et al., 2020). Investigation of the mechanisms underlying hypervirulent FAdV-4-induced IL-1β secretion will contribute to understanding of the pathogenesis of FAdV-4. Here, we investigated whether FAdV-4 activates NLRP3 inflammasome in chicken macrophage cell line HD11, and found that hypervirulent FAdV-4 stimulation activates the NLRP3 inflammasome for the first time.

The invasion of microbial pathogens induces inflammatory responses in several immune cell types including monocytes, dendritic cells, and macrophages. Macrophages are an important part of cellular immunity and have diverse functions in the tissue homeostasis and inflammatory responses to viral infection. At the early stage of viral infection, macrophages transform into M1-like macrophages which engulf the invading pathogens, release a large amount of proinflammatory cytokines and recruit other immune cells to fight against viral infections (Li et al., 2013). However, dysregulated secretion of inflammatory cytokines triggered by macrophage activities may induce inflammatory tissue damages (Labzin et al., 2019). The NLRP3 inflammasome, a vital player in the innate immunity mediating the secretion of proinflammatory cytokines IL-1β and IL-18, primarily presents in immune and inflammatory cells including macrophages. The oligomerization of NLRP3 inflammasome is initiated by DAMPs or PAMPs, forming a NLRP3, ASC and pro-Caspase-1 complex and transforming pro-Caspase-1 into its active state (Chauhan et al., 2020). Activated Caspase-1 then mediates IL-1β and IL-18 secretion by cleaving pro-IL-1β and pro-IL-18 into the biologically active forms (Muruve et al., 2008).

Caspase-1 is mainly activated in macrophage and dendritic cells. In the present study, we selected chicken bone marrow originated macrophage cell line HD11 to study the activation of NLRP3 induced by hypervirulent FAdV-4. Activation of NLRP3 inflammasome requires both type 1 priming and type 2 activating signals (Allen et al., 2009; Bauernfeind et al., 2010; Ichinohe et al., 2010; Bi et al., 2014; Lin et al., 2014; Chen et al., 2019; Zhong et al., 2020). In the priming stage, proinflammatory stimuli such as microbial products, which is FAdV-4 in our case, interact with cellular receptors to induce NLRP3 expression. Once the priming is finished, a trigger such as ATP is necessary for NLRP3 inflammasome activation (Toldo and Abbate, 2018). Since FAdV-4 alone could not induce IL-1β secretion in HD11 cells, we used hypervirulent FAdV-4 and ATP to co-stimulate HD11 cells and verified that ATP participates in the IL-1β secretion by HD11 cells in vitro as the activating signal for NLRP3. Subsequently, siRNA and recombinant plasmids for knock-down and overexpression of NLRP3 and Caspase-1 were transfected to HD11 cells followed with FAdV-4 and ATP stimulation, and it was discovered that knock-down of NLRP3 or Caspase-1 caused significant reduction of IL-1β secretion. Overexpression of NLRP3 or Caspase-1 did not significantly affect the amount of IL-1β secreted in HD11 cells stimulated with FAdV-4. This result suggested that the activation of NLRP3 and secretion of IL-1β in the chicken macrophages might be restrained in a certain degree. HD11 cells first incubated with Ac-YVAD-CHO, the activity inhibitor of Caspase-1, and then stimulated with FAdV-4 showed a significant downregulation of IL-1β secretion, indicating that chicken Caspase-1 directly mediates IL-1β secretion and its function is consistent to that in mammals (Broz and Dixit, 2016). Next, HD11 cells were treated with a low or high concentration of K^+^ before hypervirulent FAdV-4 stimulation. It was found that in the high K^+^ environment, FAdV-4 failed to activate NLRP3 inflammasome in HD11 cells. This suggested that cytosolic K^+^ efflux induces the activation of NLRP3 inflammasome in chicken macrophages infected with hypervirulent FAdV-4 under the presence of extracellular ATP. Our unpublished data indicated that FAdV-4 cannot replicate in HD11 cells, which implies FAdV-4 replication is not required for inflammasome activation in chicken macrophages, and the exact cellular receptors involved in NLRP3 activation in chicken macrophages require further study. Results in the present study demonstrated that the elevated secretion of IL-1β in the chicken macrophages stimulated with hypervirulent FAdV-4 is dependent on NLRP3 inflammasome.

Proinflammatory cytokines such as IL-1β and IL-18 secreted by monocytes and macrophages mediate the activation of differentiated lymphocytes and participates in the elimination of pathogens and release of reactive oxygen and nitrogen species (Iwasaki and Medzhitov, 2015). IL-1β recruits macrophages, monocytes, and neutrophils to sites of infection. The accumulation of IL-1β and neutrophils increases permeability of blood vessels which allows the infiltration of interstitial fluids to the site of infection and leads to cell apoptosis and tissue necrosis (Schenten and Medzhitov, 2011; Iwasaki and Medzhitov, 2015). Niu et al. observed a significant upregulation of IL-1β in the FAdV-4 infected heart and speculated that the apoptosis of cardiomyocytes induced by FAdV-4 was caused by the formation of pericardial effusion. It was also presumed that the accumulation of pericardial effusion in chickens infected with FAdV-4 was induced by the vascular exudation (Niu et al., 2019). We could hypothesize that the upregulation of IL-1β leads to increased vascular permeability which partially contributes to the vascular exudation in vivo and promotes tissue inflammatory injury in hypervirulent FAdV-4 infected chickens. The detrimental effects of excessive activity of NLRP3 and accumulation of IL-1β were studied in both human and mice (Li et al., 2013; Toldo and Abbate, 2018). Studies proved that interference with NLRP3 inflammasome activation and IL-1β secretion effectively ameliorated the brain injuries (Hoegen et al., 2011). Combined with the present study, we made the conclusion that FAdV-4 infection induced NLRP3 activation in chicken macrophages and the following upregulation of IL-1β secretion, and the accumulation of IL-1β leads to excessive inflammatory responses such as the formation of pericardial effusion, tissue inflammatory injuries, and rapid death of chicken.

To sum up, the present study confirmed that the upregulation of IL-1β in hypervirulent FAdV-4-treated chicken macrophages is dependent on NLRP3 inflammasome and Caspase-1, and FAdV-4 activates NLRP3 inflammasome through cytosolic K^+^ efflux. The present study provides new insights to the inflammatory injuries induced by FAdV-4 infection and is significant for the future investigation on the NLRP3 inflammasome activation mechanism against viral infection.
